# EMOTIONAL DISTRESS, ECONOMIC PRESSURE, AND WELL-BEING DURING COVID-19: THE ROLE OF A WARM ROMANTIC PARTNER

**DOI:** 10.1093/geroni/igad104.2663

**Published:** 2023-12-21

**Authors:** Abigail Hart, Jeenkyoung Lee, Tricia Neppl, M Heather West Greenlee

**Affiliations:** Iowa State University, Ames, Iowa, United States; Iowa State University, Ames, Iowa, United States; Iowa State University, Ames, Iowa, United States; Iowa State University, Ames, Iowa, United States

## Abstract

The COVID-19 pandemic caused health threats especially for rural older adults. We know less about how characteristics prior to the pandemic impact how older adults fare during the pandemic. Guided by the Family Stress Model, the current study examined midlife economic pressure (age 39) to emotional distress (ages 40-42) and older adult (age 70) COVID-19-related well-being (financial strain, anxiety, general health), and how these associations differ by a warm spouse. Data come from the Family Transitions Project (1989, 1990-1992, 2017, 2020) and included rural white older adults from enduring marriages (N = 265). Multiple group analysis (structural equation modeling, full information maximum likelihood) revealed significant differences between two groups divided by low and high warmth from a spouse in 2017 (low = 135, high = 130). Chi-square difference test between fixed and free model was significant (∆X2 = 9.48, ∆df = 2, p < .01), and free model had an acceptable fit to the data (X2 = 90.616, df = 56, p < .01, CFI = .97, RMSEA = .07). For low warmth group (but not high), emotional distress from economic pressure in midlife significantly predicted financial strain (b = 1.02, SE = .35, p < .01) and anxiety (b = 1.03, SE = .20, p < .001) during COVID-19. General health during COVID-19 was associated with emotional distress in midlife but did not differ by group. Our findings help understand the role of marital warmth on how earlier family stressors impact older adult well-being during historical events like COVID-19.

